# Explore impact of inhaled glucocorticoids/β₂-agonists on the oropharyngeal microbiota in asthma patients impact of GCs/β₂-agonists on oropharyngeal microbiota in asthma

**DOI:** 10.1097/MD.0000000000046483

**Published:** 2026-05-12

**Authors:** Yuliang Yang, Yanchao Liu, Runlu Wang, Jiawen Liu, Miaomiao Cui, Zheng Duan

**Affiliations:** a Department of Respiratory and Critical Care Medicine, Baoding NO.2 Central Hospital, Baoding, Hebei Province, P.R. China; b Department of Respiratory and Critical Care Medicine, The Second Hospital of Hebei Medical University, Shijiazhuang, Hebei Province, P.R. China; c Department of Respiratory and Critical Care Medicine, Shijiazhuang People’s Hospital, Shijiazhuang, Hebei Province, P.R. China; d Department of Respiratory and Critical Care Medicine, Cangzhou Central Hospital, Cangzhou City, Hebei Province, P.R. China; e Department of Respiratory and Critical Care Medicine, Hebei Provincial People’s Hospital, Shijiazhuang, Hebei Province, P.R. China.

**Keywords:** 16S rRNA sequencing, bronchial asthma, inhaled glucocorticoids/β₂-agonists, ITS sequencing, oropharyngeal microbiota

## Abstract

Asthma is a common chronic respiratory disease. Inhaled glucocorticoids (ICS) are first-line medications for asthma treatment. Studies suggest ICS use is associated with an increased risk of respiratory infections. Concurrently, multiple studies confirm a close association between respiratory microbiota and asthma, indicating that abnormal airway microbiota may be involved in the pathogenesis, progression, exacerbation, severity, disease control, and phenotypic characteristics of asthma. Whether treatment with inhaled glucocorticoids combined with long-acting β₂-agonists (ICS/LABA) affects the oropharyngeal microbiota in asthma patients, and whether this further influences treatment efficacy and prognosis, remains unclear. Using 16S rRNA amplicon sequencing and ITS gene sequencing technologies, we analyzed the bacterial and fungal microbiota in the oropharynx of 21 asthma patients who were in a clinically stable phase before and after 28 days of ICS/LABA treatment, comparing the composition and diversity of the microbial communities. Patients rinsed their mouths with water. A sterile swab was rotated twice against the posterior pharyngeal wall. ICS/LABA was taken on a fixed schedule. Significant differences were observed in the α-diversity of the oropharyngeal bacterial microbiota before and after ICS/LABA treatment in asthma patients. Post-treatment, bacterial richness and evenness significantly decreased. The relative abundances of the phylum *Candidatus* Saccharibacteria, genus *Saccharibacteria*, and species *Streptococcus oralis* were significantly lower in the post-treatment group compared to the pretreatment group. No significant differences were found in the α- or β-diversity of the oropharyngeal fungal microbiota pre- and post-treatment. However, the relative abundances of the genus *Saccharomyces* and species *Saccharomyces cerevisiae* were significantly lower post-treatment, while the relative abundances of the genus *Fusarium* and species *Thermomyces lanuginosus* were significantly higher. Association analysis revealed a decrease in *Streptococcus* abundance in the oropharynx of asthma patients after ICS/LABA treatment, suggesting a potential trend towards normalization of the bacterial microbiota, making it more similar to that of healthy individuals. The implications of the observed changes in oropharyngeal fungal microbiota following ICS/LABA treatment on asthma remain unclear and warrant further investigation.

## 1. Introduction

Asthma is a heterogeneous disease resulting from complex interactions between genetic and environmental factors,^[1]^ affecting approximately 330 million people globally,^[2,3]^ with nearly 500,000 asthma-related deaths annually. Inhaled glucocorticoids (ICS) are the first-line and primary controller medication for asthma, recommended for treating mild to severe asthma in both children and adults. While ICS improve asthma outcomes and are generally considered safe, and evidence suggests this may also be true for asthma patients,^[4,5]^ although not all studies concur.^[6]^ Oral and nasopharyngeal microbiota have been proposed as proxies for lung dysbiosis, which impacts asthma pathogenesis. Therefore, the rational use of ICS in chronic airway diseases presents complexity and challenges.

The advent of methods like 16S rRNA sequencing has confirmed the existence of a microbial ecosystem comprising bacteria, viruses, and fungi in the healthy human lung.^[7–9]^ Despite low density and bacterial load, the lung microbiota plays a crucial role in maintaining the balance between the immune system and epithelial responses.^[10]^ This microbiota is susceptible to various host factors, including genetics, lifestyle, and environment. Under certain influences, alterations and transient changes in these commensal microbes can shift them towards a more pathogenic state, potentially impacting the development and progression of diseases like chronic obstructive pulmonary disease and asthma.^[11–13]^

Increasingly, studies confirm a strong link between respiratory microbiota and asthma. Dysbiotic airway microbiota may be involved in multiple aspects of asthma, including its onset, progression, exacerbation, severity, control, and phenotype. Early life colonization by *Streptococcus pneumoniae*, *Moraxella catarrhalis*, and *Haemophilus influenzae*, as well as lower respiratory tract infections in infancy, have been linked to later development of wheezing and/or asthma in childhood.^[14–16]^ Studies show differences in airway microbiota composition between asthma patients and healthy controls.^[11,17–20]^ Asthma patients exhibit higher relative abundances of *Streptococcus*,^[18]^ *M. catarrhalis*,^[17]^ *H. influenzae*,^[11,19,21]^ and *Staphylococcus*,^[11,20]^ while *Lactobacillus*,^[22]^ *Dolosigranulum*,^[23,24]^ and *Corynebacterium*^[24]^ are associated with healthy controls. Specific respiratory microbiota signatures have been linked to asthma severity and increased exacerbation risk in both children^[25,26]^ and adults.^[27–29]^ Notably, children with stable disease showing increased *Moraxella* and decreased *Dolosigranulum*,^[24,26]^ *Corynebacterium*,^[25,26]^ and *Staphylococcus*^[25,30]^ levels are more likely to experience exacerbations. Some studies suggest dysbiosis in bronchial microbiota composition is linked to various phenotypic features in adult asthma, including airway hyperresponsiveness, airflow obstruction, response to corticosteroids, and neutrophilic inflammation.^[11,29,31–35]^

Currently, there is no consensus on defining a “healthy” lung microbiota. Similarly, the core microbiota in adult asthma remains undefined. Research on the association between airway microbiota and asthma is relatively scarce, shows inconsistent results, and predominantly focuses on the lower respiratory tract. Information on how this microbiota changes under ICS treatment is also limited. Studies indicate that ICS therapy alters the composition of the airway microbiota in asthma patients,^[33,34,36,37]^ which may influence clinical efficacy.^[33,34]^ As a topical respiratory medication, ICS/LABA has an oropharyngeal deposition rate of 10-40% (varying by device). Whether ICS/LABA treatment similarly affects the oropharyngeal microbiota in asthma patients remains unknown.

This study enrolled asthma patients before and after ICS/LABA treatment. Using 16S rRNA and ITS sequencing, we analyzed changes in the composition and diversity of oropharyngeal bacterial and fungal microbiota. We aimed to investigate the impact of ICS/LABA on this microbiota and assess the potential benefits and risks of ICS/LABA use from a microbial perspective, providing a basis for promoting more personalized medication strategies.

## 2. Materials and methods

### 2.1. Study participants

Asthma patients attending the outpatient department of the First Department of Respiratory and Critical Care Medicine at Baoding Second Central Hospital between June and November 2023, who provided written informed consent, were enrolled. The study was approved by the Ethics Committee of Baoding Second Central Hospital. All participants were informed about the study background, objectives, procedures, and risks before signing consent. All participants were in a clinically stable phase who were ICS/LABA-naive or there was a washout period before enrollment. All inhalation devices are dry powder inhalers. All participants received standard inhaler technique training and validation before entering the experiment. A dosing schedule was also provided. Exclusion criteria included: patients in the acute exacerbation period of asthma, acute respiratory infection (upper or lower) within the past 4 weeks, autoimmune diseases, malignancy, comorbid chronic obstructive pulmonary disease, lung abscess, cystic fibrosis, bronchiectasis, interstitial pneumonia or other lung diseases, antibiotic use within 4 weeks, long-term use of systemic corticosteroids, immunosuppressants, biologics, or psychiatric conditions affecting cooperation.

### 2.2. Sample collection

Oropharyngeal swabs were collected before treatment initiation and on day 28 of treatment. Patients rinsed their mouths with water. A sterile swab was rotated twice against the posterior pharyngeal wall and placed into a sterile EP tube, then stored at −20°C.

### 2.3. 16S rRNA and ITS gene sequencing for microbiota analysis

Genomic DNA was extracted from samples using the MagPure Stool DNA KF Kit B according to standard protocols. Qualified DNA (30 ng) and fusion primers were used to set up PCR reactions. PCR amplification was performed with specific parameters. Amplification products were purified using Agencourt AMPure XP beads and dissolved in Elution Buffer. Libraries were constructed and labeled. Library fragment size and concentration were assessed using an Agilent 2100 Bioanalyzer. Qualified libraries were sequenced on an appropriate platform based on insert size.

### 2.4. Data analysis

Raw paired-end reads were assembled into tags covering the hypervariable regions based on overlap. Tags were clustered into operational taxonomic units (OTUs) using USEARCH (v7.0.1090). OTU data were used to compare microbial community structures at α-diversity (within-sample) and β-diversity (between-sample) levels. α-Diversity indices (Chao1, ACE, Simpson, Shannon) were calculated. β-Diversity was assessed using both weighted and unweighted distance metrics.

### 2.5. Statistical analysis

SPSS 26.0 was used for final data analysis. Categorical data were described as frequencies (percentages). Continuous data following a normal distribution were compared using *t*-tests; non-normally distributed data were compared using the Mann–Whitney *U* test (Wilcoxon Rank-Sum Test). A *P*-value < .05 was considered statistically significant. Differences in α-diversity indices between groups were tested using the Wilcoxon Rank-Sum Test and visualized using boxplots. β-Diversity differences were assessed statistically (*P* < .05) based on distance matrices. Cluster analysis was performed based on β-diversity distances.

## 3. Results

### 3.1. Baseline characteristics

Twenty-one asthma patients (9 male [42.9%], 12 female [57.1%]) were enrolled. Mean age was 45.19 ± 15.18 years (range: 18–68 years). Four patients (19.1%) were smokers, 17 (80.9%) were nonsmokers. ICS/LABA medications included: Fluticasone/Umeclidinium/Vilanterol (1 patient, 4.8%), Budesonide/Formoterol (160/4.5 μg) (1 patient, 4.8%), Salmeterol/Fluticasone (50/500 μg) (2 patients, 9.5%), and Salmeterol/Fluticasone (50/250 μg) (17 patients, 80.9%). (Table 1)

**Table 1:** Baseline characteristics of study participants.

Characteristic	Value (n = 21)
Gender (n [%])	
Male	9 (42.9%)
Female	12 (57.1%)
Age (years, mean ± SD)	45.19 ± 15.18
Medication (n [%])	
Fluticasone/Umeclidinium/Vilanterol	1 (4.8%)
Budesonide/Formoterol 160/4.5 μg	1 (4.8%)
Salmeterol/Fluticasone 50/500 μg	2 (9.5%)
Salmeterol/Fluticasone 50/250 μg	17 (80.9%)

### 3.2. Oropharyngeal bacterial microbiota analysis

#### 3.2.1. Structure and composition

##### 3.2.1.1. Species accumulation curve

For 42 samples (21 pre, 21 post), the curve plateaued, indicating sufficient sampling depth.

##### 3.2.1.2. OTU composition

Seven hundred and sixty-two OTUs were identified. 669 OTUs were shared between pre- and post-treatment groups; 78 OTUs were unique to pretreatment, 15 to post-treatment (Venn diagram).

##### 3.2.1.3. Phylum level

Top 10 phyla pretreatment: Bacillota (43.16%), Bacteroidota (19.05%), Pseudomonadota (16.42%), Actinomycetota (11.57%), Fusobacteriota (7.30%), *Candidatus* Saccharibacteria (1.81%), Campylobacterota (0.35%), Spirochaetota (0.10%), SR1 (0.10%), Synergistota (0.02%). Top 10 phyla post-treatment: Bacillota (41.66%), Pseudomonadota (20.60%), Bacteroidota (15.06%), Actinomycetota (13.32%), Fusobacteriota (7.73%), *Candidatus* Saccharibacteria (0.91%), Campylobacterota (0.28%), Spirochaetota (0.14%), SR1 (0.12%), Synergistota (0.03%). The relative abundance of *Candidatus* Saccharibacteria was significantly lower post-treatment (*P* < .05).

##### 3.2.1.4. Genus level

Top 10 genera pretreatment: *Veillonella* (21.53%), *Prevotella* (13.22%), *Streptococcus* (12.31%), *Neisseria* (10.76%), *Rothia* (7.24%), *Fusobacterium* (4.89%), *Haemophilus* (4.61%), *Schaalia* (3.61%), *Leptotrichia* (1.89%), *Saccharibacteria* (1.81%). Top 10 genera post-treatment: *Veillonella* (23.72%), *Neisseria* (14.84%), *Prevotella* (11.08%), *Streptococcus* (10.11%), *Rothia* (9.96%), *Haemophilus* (5.28%), *Fusobacterium* (4.58%), *Schaalia* (2.94%), *Leptotrichia* (2.41%), *Saccharibacteria* (0.91%). The relative abundance of *Saccharibacteria* was significantly lower post-treatment (*P* < .05).

##### 3.2.1.5. Species level

Top 10 species pretreatment: *Veillonella dispar* (10.91%), *Streptococcus oralis* (8.75%), *Veillonella atypica* (8.03%), *Rothia mucilaginosa* (6.65%), *Prevotella melaninogenica* (6.25%), *Prevotella veroralis* (5.64%), *Fusobacterium periodonticum* (4.81%), *Haemophilus parainfluenzae* (4.23%), *Schaalia odontolytica* (2.46%), *Streptococcus parasanguinis* (1.88%). Top 10 species post-treatment: *Veillonella dispar* (13.61%), *Rothia mucilaginosa* (9.70%), *Veillonella atypica* (7.85%), *Streptococcus oralis* (6.44%), *Prevotella melaninogenica* (5.66%), *Fusobacterium periodonticum* (4.58%), *Haemophilus parainfluenzae* (4.74%), *Prevotella veroralis* (4.02%), *Streptococcus parasanguinis* (2.08%), *Schaalia odontolytica* (1.85%). The relative abundance of *Streptococcus oralis* was significantly lower post-treatment (*P* < .05).

### 3.3. Diversity analysis

#### 3.3.1. α-Diversity

Significant differences were found between pre- and post-treatment groups for sobs, ACE, coverage, Shannon, and Chao1 indices (*P* < .05). Post-treatment, bacterial richness and evenness were significantly reduced (*P* < .05).

#### 3.3.2. β-Diversity

No significant differences were found between groups using either weighted or unweighted UniFrac distances (*P* > .05), indicating no significant difference in overall bacterial community composition.

## 4. Oropharyngeal fungal microbiota analysis

### 4.1. Structure and composition

#### 4.1.1. Species accumulation curve

For 19 samples (some fungal samples may have failed QC), the curve plateaued, indicating sufficient depth.

#### 4.1.2. OTU composition

Three hundred and one OTUs were identified. One hundred and ten OTUs were shared; 71 OTUs unique to pretreatment, 120 unique to post-treatment (Venn diagram).

#### 4.1.3. Phylum level

Top 5 phyla pretreatment: Ascomycota (75.09%), Fungi_phy_Incertae_sedis (22.47%), Basidiomycota (1.01%), Chytridiomycota (0.73%), Mucoromycota (0.01%). Top 5 phyla post-treatment: Ascomycota (65.12%), Fungi_phy_Incertae_sedis (26.37%), Basidiomycota (1.01%), Chytridiomycota (0.55%), and Mucoromycota (0.06%). No significant differences at the phylum level (*P* > .05).

#### 4.1.4. Genus level

Top 10 genera pretreatment: *Saccharomyces* (26.01%), *Candida* (22.46%), Fungi_gen_Incertae_sedis (22.46%), *Paecilomyces* (17.66%), *Penicillium* (3.04%), *Aspergillus* (1.48%), *Fusarium* (1.13%), *Cladosporium* (0.68%), *Blumeria* (0.17%), *Thermomyces* (0.003%). Top 10 genera post-treatment: *Candida* (27.83%), Fungi_gen_Incertae_sedis (26.36%), *Paecilomyces* (14.03%), *Fusarium* (7.65%), *Thermomyces* (5.47%), *Penicillium* (2.83%), *Blumeria* (1.62%), *Cladosporium* (1.26%), *Aspergillus* (1.06%), *Saccharomyces* (0.021%). Post-treatment, *Saccharomyces* relative abundance was significantly lower (*P* < .05), while *Fusarium* relative abundance was significantly higher (*P* < .05).

#### 4.1.5. Species level

Top 10 species pretreatment: *Saccharomyces cerevisiae* (26.01%), Fungi_sp (22.46%), *Candida albicans* (21.94%), *Paecilomyces maximus* (17.66%), *Penicillium toxicarium* (2.51%), *Fusarium graminearum* (1.12%), *Cladosporium tenuissimum* (0.68%), Chytridiomycota_sp (0.68%), *Blumeria graminis* (0.17%), *Thermomyces lanuginosus* (0.003%). Top 10 species post-treatment: *Candida albicans* (27.49%), Fungi_sp (26.36%), *Paecilomyces maximus* (14.03%), *Fusarium graminearum* (7.54%), *Thermomyces lanuginosus* (5.47%), *Penicillium toxicarium* (1.97%), *Blumeria graminis* (1.62%), *Cladosporium tenuissimum* (1.26%), Chytridiomycota_sp (0.52%), *Saccharomyces cerevisiae* (0.02%). Post-treatment, *Saccharomyces cerevisiae* relative abundance was significantly lower (*P* < .05), while *Thermomyces lanuginosus* relative abundance was significantly higher (*P* < .05).

### 4.2. Diversity analysis

#### 4.2.1. α-Diversity

No significant differences were found between groups for sobs, ACE, coverage, Shannon, Chao1, or Simpson indices (*P* > .05), indicating no significant change in fungal richness or evenness.

#### 4.2.2. β-Diversity

No significant differences were found between groups using either weighted or unweighted UniFrac distances (*P* > .05), indicating no significant difference in overall fungal community composition.

## 5. Discussion

The human airway harbors thousands of bacterial, fungal, and viral species, collectively forming the airway microbiota. Upper and lower respiratory tract microbiota differ significantly in diversity and composition.^[38]^ The core airway microbiota in adult asthma remains undefined. Microbial communities vary across different anatomical locations within the asthmatic airway and likely play distinct roles. Research linking airway microbiota to asthma is limited, shows variable results, and predominantly focuses on the lower respiratory tract.

The human upper respiratory tract (URT) is a unique ecological niche. URT microbes, together with the mucosa, form the first line of defense against respiratory pathogens.^[39]^ Evidence links the oral microbiota to the risk of childhood atopic diseases, particularly asthma.^[22]^ Oral and nasopharyngeal microbiota have been proposed as proxies for lung dysbiosis, which impacts asthma pathogenesis.^[40]^ However, potential links between adult asthma characteristics and oral microbiota have been largely overlooked.

*Impact on Oropharyngeal Bacterial Microbiota.* Our study found that ICS/LABA treatment significantly reduced the diversity and evenness of the oropharyngeal bacterial microbiota in asthma patients. A Turkish study^[38]^ reported no difference in bacterial diversity (oral wash) between steroid-naïve and ICS-treated asthma patients. Juliana Durack et al^[41]^ found no change in oral wash bacterial diversity in mild asthma patients after 6 weeks of fluticasone treatment. Our results contradict these findings but align with an animal study by Stephanie L. Bond et al,^[42]^ showing decreased URT bacterial diversity in horses with mild asthma after 14 days of nebulized dexamethasone. These conflicting α-diversity results may stem from differences in URT sampling sites (e.g., oropharynx [swab] vs oral cavity [wash]); variations in asthma severity and control status (exacerbation vs stable); differences in ICS dose and treatment duration; and concomitant treatments (e.g., antibiotics), which drastically reduce α-diversity.

The clinical significance of reduced oropharyngeal bacterial α-diversity post-ICS/LABA is unclear. Previous studies on URT bacterial α-diversity during asthma exacerbations also show conflicting results. Some report lower α-diversity in asthmatic children vs controls,^[17,24,40]^ while others find no difference.^[17,18,43]^ Some even report higher α-diversity in asthma patients,^[44]^ associating it with longer wheezing episodes^[45]^ and loss of asthma control.^[26]^ A Spanish study^[44]^ found that ICS-treated asthma patients experiencing exacerbations had lower salivary and nasal bacterial α-diversity than non-exacerbators and exhibited greater inter-individual variation in composition, suggesting higher α-diversity may be protective against allergy and asthma. Thus, whether ICS-induced reduction in oropharyngeal bacterial diversity is protective or a potential trigger for asthma exacerbations remains unclear and requires further investigation.

Our study showed a significant decrease in *Streptococcus oralis* abundance post-treatment. *Streptococcus* is a common pathogen linked to asthma development.^[15,17]^ Cardenas et al^[46]^ suggested asymptomatic colonization with *Streptococcus*, *Haemophilus*, *Prevotella*, or *Veillonella* may increase the risk of persistent wheeze in children. *Streptococcus* abundance negatively correlates with FEV1% predicted^[20]^ and positively correlates with eosinophil percentage^[27]^ in asthma patients. Chunrong Huang et al^[47]^ found increased *Streptococcus* abundance in induced sputum of untreated asthma patients, which decreased in ICS-treated patients. Our finding of reduced oropharyngeal *Streptococcus* abundance post-ICS/LABA suggests a potential trend towards normalization of the bacterial microbiota, making it more akin to that of healthy individuals.

*Impact on Oropharyngeal Fungal Microbiota* Fungi are a significant component of the human airway microbiota. The link between fungi and asthma is long-established. *Aspergillus* species, airborne fungi, can cause allergic bronchopulmonary aspergillosis, leading to poorly controlled asthma.^[48,49]^ *Aspergillus* abundance in asthma correlates positively with impaired post-bronchodilator FEV1.^[50,51]^ *Penicillium*, *Pneumocystis*, and other indoor/outdoor fungal allergens also contribute to respiratory fungal infections and severe allergic asthma.^[52]^ Up to 30% of asthmatic children and 60% of those with severe asthma are sensitized to environmental fungi.^[53]^ Fungi clearly play important roles in asthma pathogenesis.

However, research on airway fungal microbiota is an emerging field, lagging behind bacterial studies. High-throughput sequencing now enables comprehensive characterization of commensal fungi in asthma. Studies on fungal microbiota changes in asthma are scarce, primarily focusing on the lower tract^[38,40,47,54]^; URT fungal microbiota associations are very limited. A recent small study linked URT fungal microbiota to respiratory illness in asthmatic children.^[30]^ A pediatric asthma study^[39]^ found that nasal abundance of *Malassezia globosa* was associated with lower annual exacerbation frequency and reduced risk of progression from uncontrolled asthma to severe exacerbations, suggesting URT fungi play important roles in asthma.

Our study showed significant post-treatment changes at genus (*Saccharomyces* ↓, *Fusarium* ↑) and species (*Saccharomyces cerevisiae* ↓, *Thermomyces lanuginosus* ↑) levels within the oropharyngeal fungal microbiota. The implications for asthma are unknown. Studies on ICS effects on airway fungal microbiota mainly focus on the lower tract. Chunrong Huang et al^[47]^ reported higher induced sputum abundance of *Fusarium* and *Mucor*, but lower *Wallemia*, *Alternaria*, and *Aspergillus* in ICS-treated vs untreated asthma patients, suggesting a trend towards normalization. Another Chinese study^[54]^ found non-significant reductions in induced sputum *Wallemia*, *Cladosporium*, *Penicillium*, and *Alternaria* post-ICS. Oral microbiota composition more closely resembles lung microbiota than other body sites, supporting the concept of URT microbes seeding the lungs via micro-aspiration. Thus, oral/nasopharyngeal microbiota are often used as surrogates for lung microbiota.^[44]^ However, despite some overlap, significant compositional differences exist between oral cavity and respiratory tract microbiota,^[13,40,55]^ and interactions with the immune system likely differ across mucosal sites. Therefore, the impact of ICS/LABA-induced oropharyngeal fungal changes on asthma remains unclear and requires further study.

*Limitations:* This study has limitations: small sample size; short observation period (28 days), lacking long-term ICS effect data; and need for correlating microbial changes with clinical asthma features.

In summary, this study investigated the impact of ICS/LABA on oropharyngeal bacterial and fungal microbiota in asthma patients. We demonstrated that ICS/LABA reduces bacterial diversity and *Streptococcus oralis* abundance. It also alters fungal composition, significantly decreasing *Saccharomyces cerevisiae* and increasing *Thermomyces lanuginosus*. The clinical consequences of these microbial shifts for asthma patients remain undefined and warrant further investigation.

## 6. Conclusion

Significant differences exist in the composition of oropharyngeal bacterial and fungal microbiota in asthma patients before and after ICS/LABA treatment. Post-treatment, bacterial richness and evenness significantly decreased, while fungal diversity showed no significant change. This study confirms that ICS/LABA alters the oropharyngeal microbiota, providing a reference for further exploration of asthma etiology and pathogenesis. The specific mechanisms require further clinical and basic research.

## Author contributions

**Data curation**: Runlu Wang.

**Methodology**: Jiawen Liu.

**Supervision**: Miaomiao Cui.

**Writing – original draft**: Yuliang Yang, Yanchao Liu.

**Writing – review & editing**: Yanchao Liu, Zheng Duan.

## References

[1] Cookson W. The immunogenetics of asthma and eczema: a new focus on the epithelium. Nat Rev Immunol. 2004;4:978–88. 15573132 10.1038/nri1500

[2] Global Asthma Network. The global asthma report 2018. 2018.

[3] Mukherjee M Stoddart A Gupta RP. The epidemiology, healthcare and societal burden and costs of asthma in the UK and its member nations: analyses of standalone and linked national databases. BMC Med. 2016;14:113. 27568881 10.1186/s12916-016-0657-8 PMC5002970

[4] Mckeever T Harrison TW Hubbard R Shaw D. Inhaled corticosteroids and the risk of pneumonia in people with asthma: a case-control study. Chest. 2013;144:1788–94. 23990003 10.1378/chest.13-0871

[5] Qian CJ Coulombe J Suissa S Ernst P. Pneumonia risk in asthma patients using inhaled corticosteroids: a quasi-cohort study. Br J Clin Pharmacol. 2017;83:2077–86. 28425216 10.1111/bcp.13295 PMC5555854

[6] Bansal V Mangi A Johnson MM Festic E. Inhaled corticosteroids and incident pneumonia in patients with asthma: systematic review and meta-analysis. Acta Med Acad . 2015;44:135–58. 26702909 10.5644/ama2006-124.141

[7] Segal LN Alekseyenko AV Clemente JC. Enrichment of lung microbiome with supraglottic taxa is associated with increased pulmonary inflammation. Microbiome. 2013;1:19. 24450871 10.1186/2049-2618-1-19 PMC3971609

[8] Charlson ES Bittinger K Haas AR. Topographical continuity of bacterial populations in the healthy human respiratory tract. Am J Respir Crit Care Med. 2011;184:957–63. 21680950 10.1164/rccm.201104-0655OC PMC3208663

[9] Morgan XC Huttenhower C. Chapter 12: human microbiome analysis. PLoS Comput Biol. 2012;8:e1002808. 23300406 10.1371/journal.pcbi.1002808 PMC3531975

[10] Öztürk AB Ranjan R Rani A Yazici D Bavbek S. Microbiota: the unseen players in adult asthmatic airways. Turk Thorac J. 2021;22:75–82. 33646108 10.5152/TurkThoracJ.2020.19085 PMC7919442

[11] Hilty M Burke C Pedro H. Disordered microbial communities in asthmatic airways. PLoS One. 2010;5:e8578. 20052417 10.1371/journal.pone.0008578 PMC2798952

[12] Dickson RP Huffnagle GB. The lung microbiome: new principles for respiratory bacteriology in health and disease. PLoS Pathog. 2015;11:e1004923. 26158874 10.1371/journal.ppat.1004923 PMC4497592

[13] Dickson RP Erb-downward JR Freeman CM. Bacterial topography of the healthy human lower respiratory tract. mBio. 2017;8:e02287–16. 28196961 10.1128/mBio.02287-16 PMC5312084

[14] Bisgaard H Hermansen MN Buchvald F. Childhood asthma after bacterial colonization of the airway in neonates. N Engl J Med. 2007;357:1487–95. 17928596 10.1056/NEJMoa052632

[15] Teo SM Mok D Pham K. The infant nasopharyngeal microbiome impacts severity of lower respiratory infection and risk of asthma development. Cell Host Microbe. 2015;17:704–15. 25865368 10.1016/j.chom.2015.03.008 PMC4433433

[16] Teo SM Tang HHF Mok D. Airway microbiota dynamics uncover a critical window for interplay of pathogenic bacteria and allergy in childhood respiratory disease. Cell Host Microbe. 2018;24:341–52.e5. 30212648 10.1016/j.chom.2018.08.005 PMC6291254

[17] Depner M Ege MJ Cox MJ. Bacterial microbiota of the upper respiratory tract and childhood asthma. J Allergy Clin Immunol. 2017;139:826–34.e13. 27576124 10.1016/j.jaci.2016.05.050

[18] Chun Y Do A Grishina G. Integrative study of the upper and lower airway microbiome and transcriptome in asthma. JCI Insight. 2020;5:e133707. 32161195 10.1172/jci.insight.133707 PMC7141394

[19] Boutin S Depner M Stahl M. Comparison of oropharyngeal microbiota from children with asthma and cystic fibrosis. Mediators Inflamm. 2017;2017:5047403. 29445257 10.1155/2017/5047403 PMC5763206

[20] Kim BS Lee E Lee MJ. Different functional genes of upper airway microbiome associated with natural course of childhood asthma. Allergy. 2018;73:644–52. 29052232 10.1111/all.13331

[21] Bisgaard H Hermansen MN Bonnelykke K. Association of bacteria and viruses with wheezy episodes in young children: prospective birth cohort study. BMJ. 2010;341:c4978. 20921080 10.1136/bmj.c4978 PMC2950260

[22] Dzidic M Abrahamsson TR Artacho A Collado MC Mira A Jenmalm MC. Oral microbiota maturation during the first 7 years of life in relation to allergy development. Allergy. 2018;73:2000–11. 29602225 10.1111/all.13449

[23] Song Y Hou J Kwok JSL. Whole-genome shotgun sequencing for nasopharyngeal microbiome in pre-school children with recurrent wheezing. Front Microbiol. 2022;12:792556. 35250904 10.3389/fmicb.2021.792556 PMC8889122

[24] Hou J Song Y Leung ASY. Temporal dynamics of the nasopharyngeal microbiome and its relationship with childhood asthma exacerbation. Microbiol Spectrum. 2022;10:e0012922. 10.1128/spectrum.00129-22 PMC9241764 35546575

[25] Mccauley K Durack J Valladares R. Distinct nasal airway bacterial microbiotas differentially relate to exacerbation in pediatric patients with asthma. J Allergy Clin Immunol. 2019;144:1187–97. 31201890 10.1016/j.jaci.2019.05.035 PMC6842413

[26] Zhou Y Jackson D Bachariber LB. The upper-airway microbiota and loss of asthma control among asthmatic children. Nat Commun. 2019;10:5714. 31844063 10.1038/s41467-019-13698-x PMC6915697

[27] Taylor SL Leong LEX Choo JM. Inflammatory phenotypes in patients with severe asthma are associated with distinct airway microbiology. J Allergy Clin Immunol. 2018;141:94–103.e15. 28479329 10.1016/j.jaci.2017.03.044

[28] Zhang X Zhang X Zhang N. Airway microbiome, host immune response and recurrent wheezing in infants with severe respiratory syncytial virus bronchiolitis. Pediatr Allergy Immunol. 2020;31:281–9. 31788862 10.1111/pai.13183

[29] Huang YJ Nariya S Harris JM. The airway microbiome in patients with severe asthma: associations with disease features and severity. J Allergy Clin Immunol. 2015;136:874–84. 26220531 10.1016/j.jaci.2015.05.044 PMC4600429

[30] Mccauley KE Flynn K Dimassa V. Seasonal airway microbiome and transcriptome interactions promote childhood asthma exacerbations. J Allergy Clin Immunol. 2022;150:204–13. 35149044 10.1016/j.jaci.2022.01.020

[31] Denner DR Sangwan N Becker JB. Corticosteroid therapy and airflow obstruction influence the bronchial microbiome, which is distinct from that of bronchoalveolar lavage in asthmatic airways. J Allergy Clin Immunol. 2015;137:1398–405.e3. 26627545 10.1016/j.jaci.2015.10.017 PMC4860110

[32] Durack J Boushey HA Lynch SV. Airway microbiota and the implications of dysbiosis in asthma. Curr Allergy Asthma Rep. 2016;16:52. 27393699 10.1007/s11882-016-0631-8

[33] Durack J Lynch SV Nariya S. Features of the bronchial bacterial microbiome associated with atopy, asthma, and responsiveness to inhaled corticosteroid treatment. J Allergy Clin Immunol. 2017;140:63–75. 27838347 10.1016/j.jaci.2016.08.055 PMC5502827

[34] Goleva E Jackson LP Harris JK. The effects of airway microbiome on corticosteroid responsiveness in asthma. Am J Respir Crit Care Med. 2013;188:1193–201. 24024497 10.1164/rccm.201304-0775OC PMC3863730

[35] Huang YJ Nelson CE Brodie EL. Airway microbiota and bronchial hyperresponsiveness in patients with suboptimally controlled asthma. J Allergy Clin Immunol. 2011;127:372–81.e1. 21194740 10.1016/j.jaci.2010.10.048 PMC3037020

[36] Sharma A Laxman B Naureckas ET. Associations between fungal and bacterial microbiota of airways and asthma endotypes. J Allergy Clin Immunol. 2019;144:1214–27.e7. 31279011 10.1016/j.jaci.2019.06.025 PMC6842419

[37] Zhang Q Cox M Liang Z. Airway microbiota in severe asthma and relationship to asthma severity and phenotypes. PLoS One. 2016;11:e0152724. 27078029 10.1371/journal.pone.0152724 PMC4831690

[38] Oğuzülgen K Öztürk AB Baccecoğlu A. Inhaler steroid use changes oral and airway bacterial and fungal microbiome profile in asthma patients. Int Arch Allergy Immunol. 2024;185:10–9. 37844548 10.1159/000531866

[39] Yuan H Liu Z Dong J. The fungal microbiome of the upper airway is associated with future loss of asthma control and exacerbation among children with asthma. Chest. 2023;164:302–13. 37003356 10.1016/j.chest.2023.03.034 PMC10477953

[40] Perez-garcia J González-carracedo M Espuela-ortiz A. The upper-airway microbiome as a biomarker of asthma exacerbations despite inhaled corticosteroid treatment. J Allergy Clin Immunol. 2023;151:706–15. 36343772 10.1016/j.jaci.2022.09.041

[41] Durack J Christian LS Nariya S. Distinct associations of sputum and oral microbiota with atopic, immunologic, and clinical features in mild asthma. J Allergy Clin Immunol. 2020;146:1016–26. 32298699 10.1016/j.jaci.2020.03.028 PMC7554083

[42] Bond SL Workentine M Hundt J Gilkerson JR Léguillette R; UCVM Class of 2019. Effects of nebulized dexamethasone on the respiratory microbiota and mycobiota and relative equine herpesvirus-1, 2, 4, 5 in an equine model of asthma. J Vet Intern Med. 2020;34:307–21. 31793692 10.1111/jvim.15671 PMC6979091

[43] Thorsen J Stokholm J Rasmussen MA. Asthma and wheeze severity and the oropharyngeal microbiota in children and adolescents. Ann Am Thorac Soc. 2022;19:2031–43. 35904980 10.1513/AnnalsATS.202110-1152OC

[44] Van Beveren GJ De Steenhuijsen Piters WAA Boeschoten SA. Nasopharyngeal microbiota in children is associated with severe asthma exacerbations. J Allergy Clin Immunol. 2024;153:1574–85. 38467291 10.1016/j.jaci.2024.02.020

[45] Thorsen J Stokholm J Rasmussen MA. The airway microbiota modulates effect of azithromycin treatment for episodes of recurrent asthma-like symptoms in preschool children: a randomized clinical trial. Am J Respir Crit Care Med. 2021;204:149–58. 33730519 10.1164/rccm.202008-3226OC

[46] Marsland BJ Gollwitzer ES. Host-microorganism interactions in lung diseases. Nat Rev Immunol. 2014;14:827–35. 25421702 10.1038/nri3769

[47] Huang C Yu Y Du W. Fungal and bacterial microbiome dysbiosis and imbalance of trans-kingdom network in asthma. Clin Transl Allergy. 2020;10:42. 33110490 10.1186/s13601-020-00345-8 PMC7583303

[48] Agarwal R Gupta D. Severe asthma and fungi: current evidence. Med Mycol. 2011;49:S150–7. 20662637 10.3109/13693786.2010.504752

[49] Woolnough K Fairs A Pashley CH Wardlaw AJ. Allergic fungal airway disease: pathophysiologic and diagnostic considerations. Curr Opin Pulm Med. 2015;21:39–47. 25415407 10.1097/MCP.0000000000000129

[50] Van Woerden HC Gregory C Brown R. Differences in fungi present in induced sputum samples from asthma patients and non-atopic controls: a community based case control study. BMC Infect Dis. 2013;13:69. 23384395 10.1186/1471-2334-13-69 PMC3570489

[51] Agbetile J Fairs A Desai D. Isolation of filamentous fungi from sputum in asthma is associated with reduced post-bronchodilator FEV1. Clin Exp Allergy. 2012;42:782–91. 22515394 10.1111/j.1365-2222.2012.03987.x PMC3509218

[52] Singh M Paul N Singh S Nayak GR. Asthma and fungus: role in allergic bronchopulmonary aspergillosis (ABPA) and other conditions. Indian J Pediatr. 2018;85:899–904. 29549557 10.1007/s12098-018-2646-8

[53] Baxi SN Portnoy JM Larenas-linnemann D Phipatanakul W; Environmental Allergens Workgroup. Exposure and health effects of fungi on humans. J Allergy Clin Immunol Pract. 2016;4:396–404. 26947460 10.1016/j.jaip.2016.01.008 PMC4861659

[54] Huang C Ni Y Du W Shi G. Effect of inhaled corticosteroids on microbiome and microbial correlations in asthma over a 9-month period. Clin Transl Sci. 2022;15:1723–36. 35514165 10.1111/cts.13288 PMC9283747

[55] Durack J Huang YJ Nariya S. Bacterial biogeography of adult airways in atopic asthma. Microbiome. 2018;6:104. 29885665 10.1186/s40168-018-0487-3 PMC5994066

